# Aflatoxins and safe storage

**DOI:** 10.3389/fmicb.2014.00158

**Published:** 2014-04-10

**Authors:** Philippe Villers

**Affiliations:** GrainPro, Inc.Concord, MA, USA

**Keywords:** post harvest, aflatoxin, hermetic, safe storage, grain storage, Cocoons™, pesticide free, Ultra Hermetic

## Abstract

The paper examines both field experience and research on the prevention of the exponential growth of aflatoxins during multi-month post-harvest storage in hot, humid countries. The approach described is the application of modern safe storage methods using flexible, Ultra Hermetic™ structures that create an unbreatheable atmosphere through insect and microorganism respiration alone, without use of chemicals, fumigants, or pumps. Laboratory and field data are cited and specific examples are given describing the uses of Ultra Hermetic storage to prevent the growth of aflatoxins with their significant public health consequences. Also discussed is the presently limited quantitative information on the relative occurrence of excessive levels of aflatoxin (>20 ppb) before vs. after multi-month storage of such crops as maize, rice, and peanuts when under high humidity, high temperature conditions and, consequently, the need for further research to determine the frequency at which excessive aflatoxin levels are reached in the field vs. after months of post-harvest storage. The significant work being done to reduce aflatoxin levels in the field is mentioned, as well as its probable implications on post-harvest storage. Also described is why, with some crops such as peanuts, using Ultra Hermetic storage may require injection of carbon dioxide, or use of an oxygen absorber as an accelerant. The case of peanuts is discussed and experimental data is described.

## Introduction

Aflatoxins (*Aspergillus flavus and Aspergillus parasiticus*) are widely recognized as a major health problem, especially in hot, humid countries. This is a particular serious problem in such crops as maize, rice, peanuts, tree nuts, and dried fruits. Aflatoxin production normally occurs in the field, particularly when stimulated by drought, stress, and high temperatures or during prolonged drying. Aflatoxin-producing molds grow exponentially in conventional multi-month storage as a result of a combination of heat and high humidity (Hell et al., 2010).

**Figure 1:**
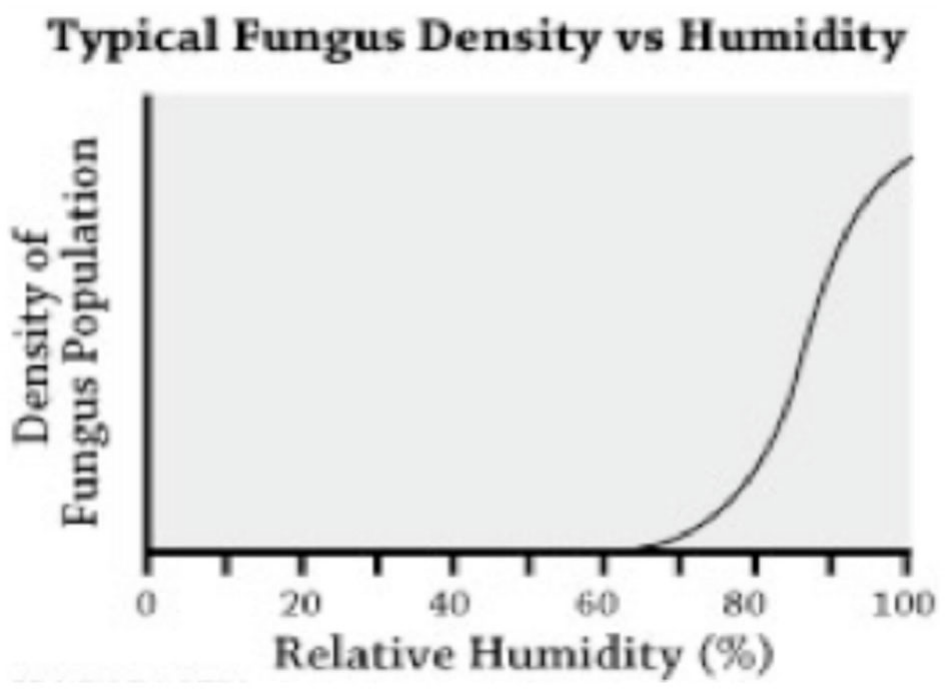
**Typical fungus density vs. humidity**.

Figure 1 (Villers et al., 2008) shows the graphic relationship between mold growth and relative humidity at equilibrium. It explains the mold growth during storage, when sustained relative humidity is beyond 65%. Table 1 shows the optimum conditions and ranges for *Aspergillus* growth and therefore aflatoxin development. When temperatures are below 65 degrees F, and the moisture of the maize is below 12–13%, development of the fungus usually stops (Sumner and Lee, 2012).

**Table 1:** **Conditions favoring *Aspergillu*s *flavus* development**.

**Factor**	**Optimum**	**Range**
Temperature	86°F	80–110°F
Relative humidity	85%	62–99%
Kernel moisture	18%	13–20%

*Table shows the optimum conditions for Aspergillus growth and aflatoxin development. When temperatures are below 65°F and the moisture of the corn is below 12–13%, development of the fungus usually stops*.

**Figure 2:**
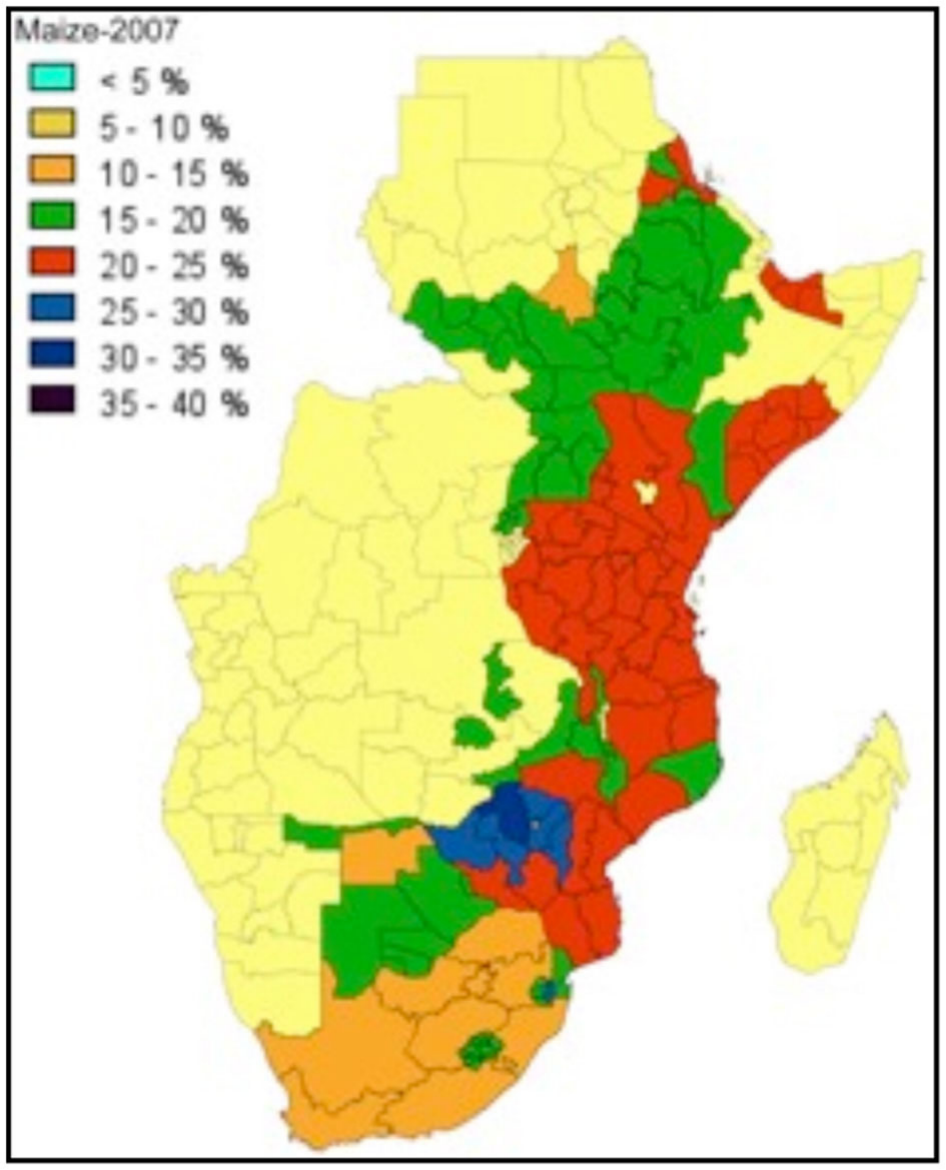
**2011 World Bank Report on post-harvest losses of maize in East Africa**.

In East Africa post-harvest losses in maize, as shown in Figure 2, often reach 25%, or more (World Bank Report, 2011).

## Aflatoxin effects

Dr. J. H. Williams of the University of Georgia, USA, cites a survey of local African markets showing that 40% of the commodities found there exceeded permissible aflatoxin levels (in excess of the international standard of 10–20 ppb) and that an estimated 4.5 billion people in developing countries are at risk of uncontrolled or poorly controlled exposure to aflatoxins, and up to 40% of commodities in local African markets exceed allowable levels of aflatoxins in foods (Williams, 2011).

It is known that high aflatoxin levels in the bloodstream depresses the immune system, thereby facilitating cancer, HIV, and stunting the growth of children. A cross-sectional study conducted in Ghana and cited by Dr. Williams shows that immune systems of recently HIV-infected people are significantly modified if they have above-median levels of natural exposures to aflatoxins (Williams et al., 2004). Referring to another study, Dr. Williams notes, “People with a high aflatoxin biomarker status in the Gambia and Ghana were more likely to have active malaria” (Williams, 2011).

In 2014, the Global Forum for Innovations in Agriculture (GFIA) convened a high level meeting in Abu Dhabi, UAE, on revolutionizing global agriculture through innovations. Frank Rijsberman, the CEO of the CGIAR Consortium, in his report based on a Benin study (Gong et al., 2004) on the post-weaning exposure to aflatoxin, concludes that aflatoxins have impaired growth in children and are costing African farmers over $450 million USD per year in lost exports (Rijsberman, 2014). In 2010, 10% of the Kenyan maize crop was condemned because of excessive aflatoxin levels. One of the laboratories in Kenya that year tested 130 maize samples out of which only 47 samples had aflatoxin levels less than 10 ppb. The highest level of aflatoxin recorded in that year was 830 ppb (FAO, 2011).

Excessive aflatoxin levels also cause failure to thrive (or even death) in farm animals such as chickens and turkeys. According to Dr. Oladele Dotun, a Veterinarian at the Animal Care Laboratory in Nigeria, research has shown that aflatoxins cause infertility, abortions, and delayed onset of egg production in birds as well as sudden losses in egg production in actively laying birds. Furthermore, loss of appetite, skin discoloration, or even yellowish pigmentation on skin can be observed in fish (Oladele, 2014).

An increasing amount of scientific research has been devoted to learning more about aflatoxin growth problems and possible solutions, including using genetically modified or hybridized seeds formulated for mold resistance or through use of products such as AflaSafe, now used in Africa. AflaSafe's “biological approach” uses a closely related, non-aflatoxin-producing mold to out-compete the aflatoxin-producing molds. In temperate climates, aflatoxin problems have been controlled largely with ventilation during cooler nights and through lower winter temperatures (CAES, 2014).

**Figure 3:**
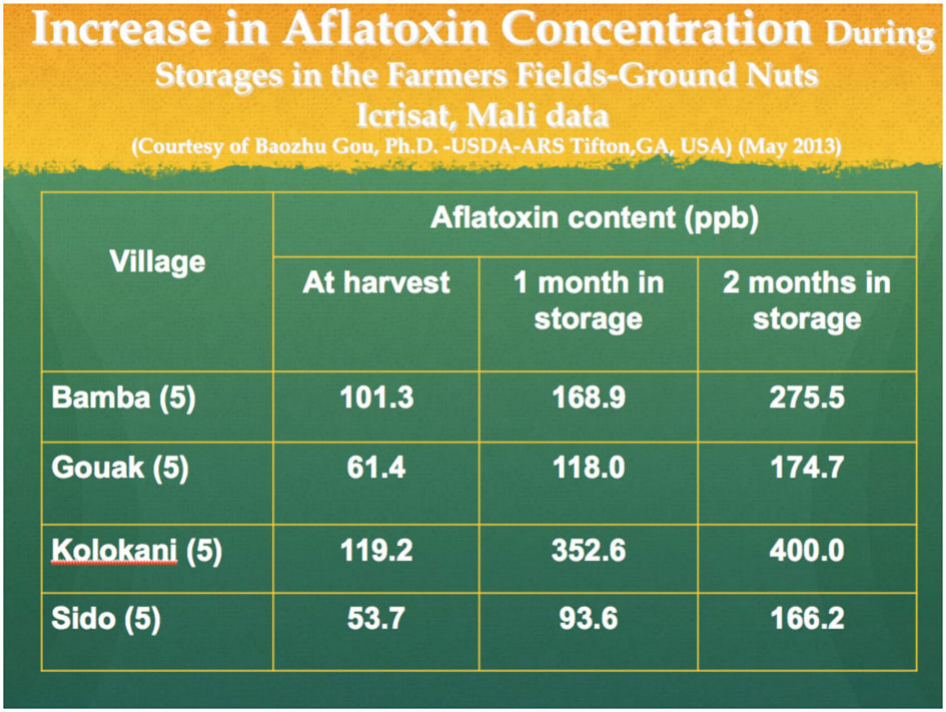
**Increase in aflatoxin concentration during storage in the farmers' fields**.

A neglected part of the aflatoxin problem has been the rapid growth of post-harvest aflatoxin levels when stored for prolonged periods in the conditions of high temperature and relative humidity above 65% found commonly in all tropical climates (See Figure 1). In the tropics, field studies by Icrisat in Mali show that conventional storage for more than 2 months causes rapid growth of aflatoxins (Waliyar et al., 2002) (Figure 3).

In Guatemala, a field survey of maize sold in rural markets by Martinez-Herrera in 1968 found that considerable contamination by several fungi, including *Aspergillus* species were frequently present. His evidence suggests that maximum aflatoxin contamination of maize in Guatemala occurs during the rainy season. Maize samples analyzed 20 days after harvest had levels of 130 μg aflatoxin per kg of total maize. The same samples analyzed 60 days later showed rapid increase, up to 1 680 μg per kg (FAO, 1992).

This data, as well as data from several other studies, strongly indicate the need to dry maize before storage. Drying is an important step in ensuring good quality grain that is free of fungi and microorganisms and that has desirable quality characteristics for marketing and final use. Diverse drying systems and equipment are available that use various sources of energy, including solar energy. Choice of system requires consideration of a number of factors: temperature and air velocity, rate of drying, drying efficiencies, kernel quality, air power, fuel source, fixed costs, and management (FAO, 1992).

An interesting question is what happens to aflatoxin growth in Ultra Hermetic Storage™ following the use of biological controls such as AflaSafe, which introduces non-aflatoxin-producing molds to compete with aflatoxin-producing molds. However, biological competition between mold populations alone will not totally eliminate aflatoxin mold prior to storage. We believe that storage under tropical conditions in classical containers such as silos, bags, and tarps will continue to produce the exponential aflatoxin growth shown in Figure 1, probably from a lower starting population.

## Chemical approaches to post-harvest control

Many chemical and additive methods for controlling aflatoxin growth have been proposed, but used only to a limited extent. For instance, as noted by Lunven at FAO, “Chakrabarti showed that aflatoxin levels could be reduced to less than 20 ppb using separate treatments with 3% hydrogen peroxide, 75% methanol, 5% dimethylamine hydrochloride, or 3% perchloric acid. These treatments, however, induced losses in weight and also in protein and lipids. Other methods include the use of carbon dioxide plus potassium sorbate and the use of sulphur oxide.” Lunven also noted another process that had received some attention, namely the use of calcium hydroxide, a chemical used for lime cooking of maize. Regarding this process, Lunven stated, “Studies have shown a significant reduction in aflatoxin levels, although the extent of reduction is related to the initial levels. Feeding tests with moldy maize treated with calcium hydroxide have shown a partial restoration of its nutritional value” (FAO, 1992).

However, to date, no chemical or additive method has gained general acceptance. Concerns about using chemicals or additives is still growing, encouraging the expanded use of methods that have no contamination potential.

## Safe storage using ultra hermetic airtight containers

Ultra Hermetic Storage™ is in use now at a varying scale in 103 countries. It has been shown that WHEN sufficiently airtight, it drastically inhibit mold growth and hence aflatoxin expression. Since molds need oxygen and high humidity, the natural respiration of the contained insects and other microorganisms, and sometimes respiration of the commodity itself, will use up the available oxygen faster than any residue leakage. After 10 days to 2 weeks at room temperature or above, this results in an unbreatheable atmosphere–typically 3% oxygen and 15% carbon dioxide. This does not kill the molds, but as shown in Table 2, in the case of peanuts, the unbreatheable atmosphere arrests mold development of Colony Forming Units (CFUs) so that even after several months, the levels of mold products, and therefore aflatoxins do not rise significantly. The same table shows inhibition of Free Fatty Acids (FFAs), which cause rancidity (Navarro et al., 2012).

**Table 2:** **FFA and CFU mold levels in peanuts**.

**Moisture content (%)**	**Test parameters**	**Initial**	**After 90 days**				
			**Hermetic clean peanuts**	**Hermetic with 3% broken peanuts**	**CO**_2_** with 3% broken peanuts**	**Control clean peanuts**	**Control with 3% broken peanuts**
**7**	**% Moisture content**	5.97 ± 0.03	6.80 ± 0.20	7.20 ± 0.21	6.60 ± 0.40	6.33 ± 0.53	6.60 ± 0.26
	**FFA (% oleic acid)**	0.36 ± 0.01	0.63 ± 0.53	0.70 ± 0.17	0.43 ± 0.07	0.57 ± 0.03	1.50 ± 0.12
	**Aflatoxin (μg/kg)**	<0.3	<0.3	<0.3	<0.3	<0.3	<0.3
	**CFU molds**	3*10_2_	1.8*10_3_ ± 1.2*10_3_	1.7*10_3_ ± 7*10_2_	9.7*10_1_ ± 28	1.3*10_4_ ± 9*10_3_	4*10_4_ ± 3*10_3_
**8**	**% Moisture content**	7.53 ± 0.07	6.87 ± 0.15	6.37 ± 0.2	7.10 ± 0.32	6.63 ± 0.19	7.30 ± 0.17
	**FFA (% oleic acid)**	0.42 ± 0.09	0.67 ± 0.17	2.13 ± 0.07	0.77 ± 0.03	2.57 ± 0.47	4.00 ± 0.42
	**Aflatoxin (μg/kg**)	<0.3	<0.3	<0.3	<0.3	<0.3	<0.3

Martin Gummert, post-harvest rice specialist at the International Rice Research Institute (IRRI), wrote about storage of rice and rice seed, “Hermetic Storage of rice is becoming increasingly popular across Asia, and for good reason—as well as being transportable, it is better than air-conditioned storage and almost as good as a cold room, at a fraction of the cost” (Villers and Gummert, 2009).

## The special case of peanuts

Peanuts often are often contaminated with aflatoxins before storage, but (unlike most grains) may take as long as 30 days or so to reach a 3% oxygen level in Ultra Hermetic storage (Navarro et al., 2012). This is too long a period to prevent major increase of aflatoxin levels. For this reason, one of two forms of accelerant is used. For field operation and portable bags, an oxygen-absorbing sachet weighing 65 g per 69 kg capacity is sufficient. For large storage units or volume production, injecting carbon dioxide to drive out the air is adequate. Table 2 shows mold growth densities measured in CFUs after 90 days (Navarro et al., 2012). The table shows two orders of magnitude difference in mold density for crops stored in conventional (the Control) vs. hermetic storage, using carbon dioxide injection. Hermetic storage, even without injected carbon dioxide, still shows a five-fold improvement vs. the Control. Unfortunately, in this particular study, aflatoxin-producing molds turned out to be largely absent in the mold mix, and therefore the data of Table 2 does not allow direct measurement of the growth of aflatoxin, as distinct from all molds (CFU). Table 2 shows the significant improvement with hermetic storage alone and even greater improvement with the early addition of CO_2_ in controlling the growth of rancidity-producing FFAs.

## An unsolved question

Needing further study is the question of exactly how frequently aflatoxin levels exceed the international standard of 10–20 ppb at initial storage time vs. after long-term storage. The unanswered question is how frequently initial tolerable levels of aflatoxins become intolerable after improper drying and or improper post-harvest storage. We believe it is quite likely, that the levels of aflatoxin in grains registering above 10–20 ppb in peanuts before storage may well be relatively infrequent but only exceed the international safety standard after long-term storage. The absence of a definitive answer to this question may well contribute to scientific attention still being devoted almost entirely to the problem of controlling aflatoxin in the field. To date, the demonstrated use of Ultra Hermetic storage, although widespread and available, has not yet resulted in much scientific work being focused on existing ways of preventing aflatoxin growth post-harvest and in longer-term storage. We believe such study is overdue.

## Available forms of ultra hermetic airtight storage

**Figure 4:**
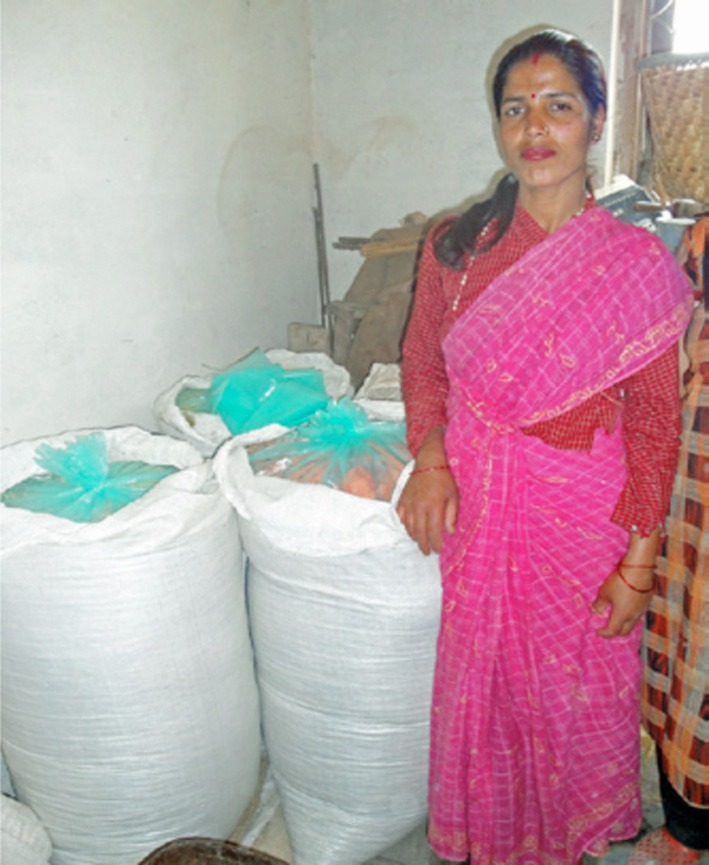
**Nepalese woman with SuperGrainbags, Mulpani Village near Kathmandu, Nepal**.

**Figure 5:**
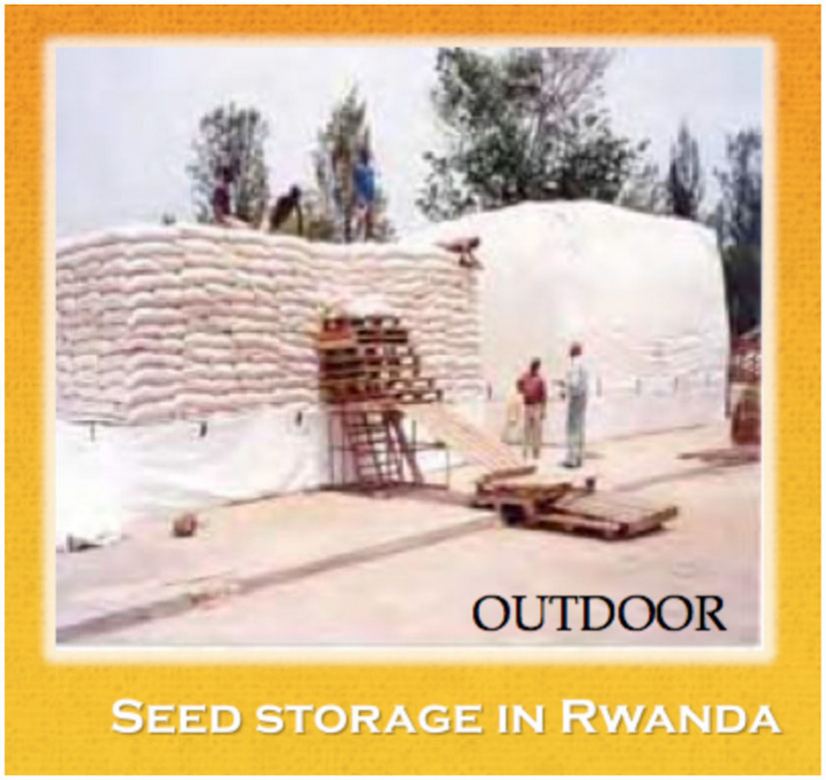
**Outdoor seed storage in Rwanda**.

Ultra-Hermetic™ (airtight) enclosures without pesticides or fumigants use the respiration of infesting insects and other microflora as their “engines” to produce an unbreatheable atmosphere. Available sizes of such airtight plastic enclosures vary over a wide range depending on the application. They range from the patented man-portable SuperGrainbags™, made from thin (0.078 mm) coextruded, multi-layer plastic with capacities from 25 kg to 2.8 tons (Figure 4), to flood- and tsunami-resistant Cocoons™ made from 0.8 mm PVC with capacities of 5- to 1000-tonnes (Figure 5). Their massive air and watertight bulk and geometry help explain their survival when full in several Philippine typhoons and in one flood.

Cocoons are also highly rodent-resistant, as a result of intentionally stretching their tough exterior surface to prevent rodents from gaining a tooth hold. Cocoons can be erected without specialized tools or equipment and can be rapidly installed either directly on the ground or on pavement, either indoors or outdoors, and have a 10–15 year useful life.

The SuperGrainbag, man-portable Ultra Hermetic storage shown in Figure 4 has permeability to oxygen of only 3 cc/m^2^/per day, 500 times more airtight than ordinary plastics such as polyethylene. This results from the use of a proprietary barrier layer inside its 3-layer, coextruded design.

Figure 5 shows 100-tonne Cocoons enclosing bags of grains stacked on the bottom of the Cocoon. After the bags are stacked, the Cocoon is closed by mounting its upper section over the pile. Then the two sections are zipped together with an airtight zipper, originally designed for astronauts.

**Figure 6:**
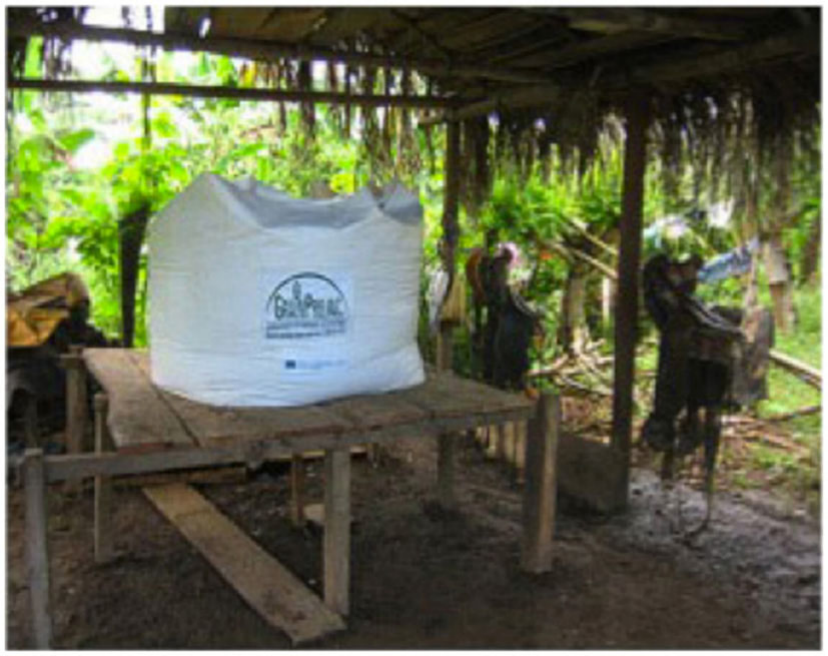
**GrainSafe in Guatemala storing maize**.

Other designs, such as the 1- to 2-tonne capacity GrainSafe™ shown in Figure 6, are used for storing bulk grain, and are designed for continuous in-and-out access while maintaining hermeticity and avoiding empty headspace when partly empty.

**Figure 7:**
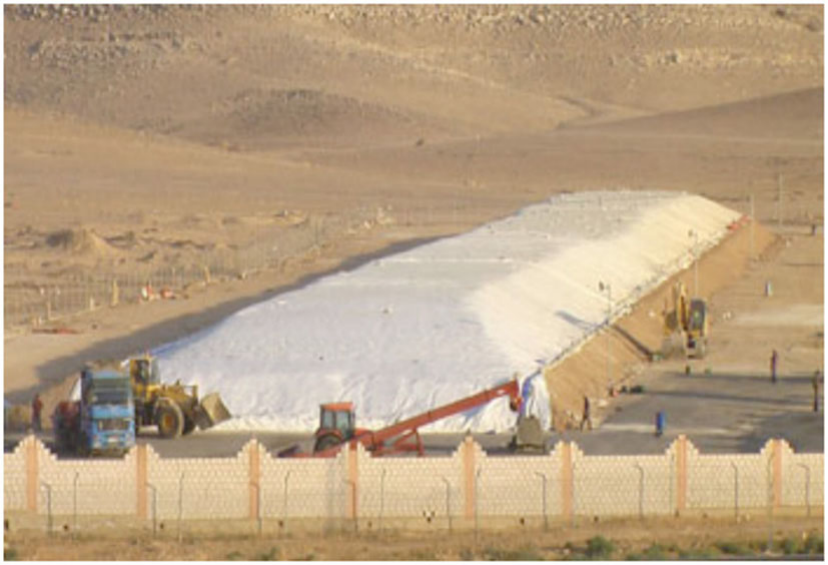
**Bunker in Jordan storing wheat**.

The largest existing Ultra Hermetic storage units are known as Hermetic Bunkers™ and store 10,000- to 20,000-tonnes (Figure 7) (Navarro et al., 1993). They are designed for multi-year strategic storage. Hermetic Bunkers are used for up to 5-year storage of barley, maize or wheat, such as in Cyprus, Jordan, and Israel (Navarro et al., 1993).

Rice and other grain seeds now are among the leading commodities stored in Ultra Hermetic enclosures instead of air-conditioned or refrigerated facilities (Villers and Gummert, 2009).

## Safe solar drying

**Figure 8:**
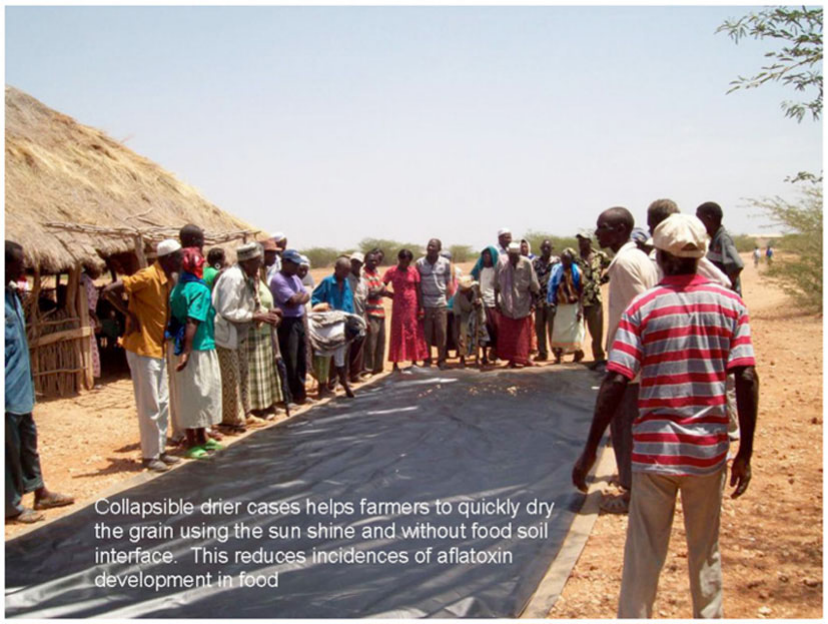
**Collapsible solar dryer in Ghana**.

All forms of longer-term storage historically have required proper drying before storage. Various strategies have been used, but in most developing countries the capital and consumption costs of using non-renewable fuels are unaffordable, and so solar drying is used. However, much solar drying is done on paved patios, paved roads, or even dirt roads, with no protection from rewetting when it rains. The Collapsible Solar Dryer (CDC)™ (Figure 8) has the special property of being manually reclosed on itself when it rains, creating a rainproof cover like a suitcase lid that keeps the seeds or grains dry until the rain stops.

## Conclusion

Programs to control aflatoxin-producing molds in the field through improved seeds or biological control have yielded encouraging results, but they do not protect commodities from exponential aflatoxin growth in post-harvest storage under hot, humid conditions, which in many cases may be the dominant growth factor. Ultra Hermetic storage is an available, important answer to keeping aflatoxin levels at (or below) the International Standards of a maximum of 10- to 20-ppm, now so often exceeded in hot, humid climates. It is already widely proven in the field but not yet utilized on a sufficient scale. To date, the importance of post-harvest protection against aflatoxin growth remains not widely understood by the scientific community. Existing literature on aflatoxin control focuses on means of reducing aflatoxin levels during the growing period, and more work is needed to prove the full public health significance of aflatoxin growth during conventional drying and in non-hermetic, post-harvest storage vs. levels of aflatoxin already existing by harvest time.

### Conflict of interest statement

The author declares that the research was conducted in the absence of any commercial or financial relationships that could be construed as a potential conflict of interest.

## References

[B2] CAES (2014). Reducing Aflatoxins in Corn During Harvest. Atlanta, GA: CAES Publication, University of Georgia, B 1231

[B3] FAO (1992). Maize in Human Nutrition. Food and Nutrition Series, No. 25, Chapter 3, Rome: Food Policy and Nutrition Division

[B4] FAO (2011). Situation analysis: improving food safety in the maize value chain in Kenya, in Report prepared for FAO by Prof. Erastus Kang'ethe College of Agriculture and Veterinary Science, University of Nairobi

[B5] Gong Y. Y. Hounsa A. Egal S. Turner P. C. Sutcliffee A. E. Hall A. J. (2004). Post weaning exposure to aflatoxin results in impaired child growth: a longitudinal study in Benin, West Africa. Environ. Health Perspect. 112, 1334–1338 10.1289/ehp.6954 15345349 PMC1247526

[B6] Hell K. Mutegi C. Fandohan P. (2010). Aflatoxin control and prevention strategies in maize for Sub-Saharan Africa, in 10th International Working Conference on Stored Product Protection, Vol. 2 (Estoril), 534–540; *Green. J. Agricult. Sci* 2, 280, October 2012.

[B7] Navarro H. Navarro S. Finkelman S. (2012). Hermetic and modified atmosphere storage of shelled peanuts to prevent free fatty acid and Aflatoxin formation, in Proceedings of the Conf. Int. Org. Biol. Integrated Control of Noxious Animals and Plants (IOBC), Work Group on Integrated Prot. Stored Prod. Bull. (Volos).

[B8] Navarro S. Varnava A. Donahaye E. (1993) Preservation of grain in hermetically sealed plastic liners with particular reference to storage of barley in Cyprus, in Proceedings International Conference on Controlled Atmosphere and Fumigation in Grain Storages, Winnipeg, Canada, June 1992, eds Navarro S. Donahaye E. (Jerusalem: Caspit Press Ltd.), 223–234

[B9] Oladele D. (2014). “The effects of aflatoxins on animals,” Partnership for Aflatoxin Control in Africa (Meridian Institute, Washington, DC), Aflatoxin Partnership Newsletter, Vol. II (Accessed, February 2014), 4

[B10] Rijsberman F. (2014). PACA (Partnership for Aflatoxin Control in Africa) presents at the global forum for innovations: middle east and Africa focus (Meridian Institute, Washington, DC), Aflatoxin Partnership Newsletter, Vol. II (Accessed February, 2014).

[B12] Sumner P. E. Lee D. (2012). Reducing Aflatoxin in Corn During Harvest and Storage. Atlanta, GA: The University of Georgia, Georgia College of Agriculture and Environmental Sciences

[B13] Villers P. Gummert M. (2009). Seal of approval. Rice Today (Los Banos, CA) 8, 26–27

[B14] Villers P. Navarro S. DeBruin T. (2008). Development of hermetic storage technology in sealed flexible storage structures, in CAF 2008 Conference Paper (Chengdu).

[B16] Waliyar F. Natre B. R. Traore A. Diarra B. Kodio O. Lava Kumar P. (2002). Pre and Postharvest Management of Aflatoxin Contamination in Groundnut. Bamako: ICRISAT (International Crops Research Institute for the Semi-Arid Tropics)

[B15] Williams J. H. (2011). Aflatoxin as a Public Health Factor in Developing Countries and its Influence on HIV and Other Diseases. Peanut Collaborative Research Support Program, University of Georgia, World Bank Report #60371-AFR, 1–95

[B17] Williams J. H. Phillips T. D. Jolly P. E. Stiles J. K. Jolly C. M. Aggarwal D. (2004). Human aflatoxicosis in developing countries: a review of toxicology, exposure, potential health consequences, and interventions. Am. J. Clin. Nutr. 80, 1106–1122 15531656 10.1093/ajcn/80.5.1106

